# *Desulfonatronovibrio halophilus* sp. nov., a novel moderately halophilic sulfate-reducing bacterium from hypersaline chloride–sulfate lakes in Central Asia

**DOI:** 10.1007/s00792-012-0440-5

**Published:** 2012-04-10

**Authors:** D. Y. Sorokin, T. P. Tourova, B. Abbas, M. V. Suhacheva, G. Muyzer

**Affiliations:** 1 Winogradsky Institute of Microbiology, Russian Academy of Sciences, Prospect 60-let Octyabrya 7/2, 117811 Moscow, Russia; 2 Environmental Biotechnology Group, Department of Biotechnology, Delft University of Technology, Delft, The Netherlands; 3 Bioengineering Centre, Russian Academy of Sciences, Prospect 60-let Octyabrya 7/1, 117811 Moscow, Russia; 4 Present Address: Department of Aquatic Microbiology, Institute for Biodiversity and Ecosystem Dynamics, University of Amsterdam, Amsterdam, The Netherlands

**Keywords:** Sulfate-reducing bacteria (SRB), *Desulfonatronovibrio*, Hypersaline lakes, Halophilic

## Abstract

Four strains of lithotrophic sulfate-reducing bacteria (SRB) have been enriched and isolated from anoxic sediments of hypersaline chloride–sulfate lakes in the Kulunda Steppe (Altai, Russia) at 2 M NaCl and pH 7.5. According to the 16S rRNA gene sequence analysis, the isolates were closely related to each other and belonged to the genus *Desulfonatronovibrio*, which, so far, included only obligately alkaliphilic members found exclusively in soda lakes. The isolates utilized formate, H_2_ and pyruvate as electron donors and sulfate, sulfite and thiosulfate as electron acceptors. In contrast to the described species of the genus *Desulfonatronovibrio*, the salt lake isolates could only tolerate high pH (up to pH 9.4), while they grow optimally at a neutral pH. They belonged to the moderate halophiles growing between 0.2 and 2 M NaCl with an optimum at 0.5 M. On the basis of their distinct phenotype and phylogeny, the described halophilic SRB are proposed to form a novel species within the genus *Desulfonatronovibrio*, *D. halophilus* (type strain HTR1^T^ = DSM24312^T^ = UNIQEM U802^T^).

**Electronic supplementary material:**

The online version of this article (doi:10.1007/s00792-012-0440-5) contains supplementary material, which is available to authorized users.

## Introduction

Hypersaline intra-continental (*athalassohaline*) salt lakes represent an extreme type of habitats where halophilic prokaryotes are a dominant form of life (Oren 2002). The sulfur cycle is definitely active in the sediments of these lakes, judging from the presence of diverse population of halophilic sulfur-oxidizing bacteria (Sorokin 2008) and the apparent sulfidogenic activity manifested by the presence of high concentrations of FeS/HS^−^ in the top sediment layer and measurable activity of sulfate reduction, albeit at salinities much lower than salt saturation (Brandt et al. 2001; Sørensen et al. 2004; Waldron et al. 2007).

Our recent study on sulfidogenesis in anoxic sediments of hypersaline chloride–sulfate lakes in Kulunda Steppe (Altai, Russia) (Sorokin et al. 2012) demonstrated the presence of several groups of moderately halophilic SOB there, most of which were highly similar to the phylotypes found previously in the Great Salt Lake in Utah (USA) (Brandt et al. 1999, 2001; Jakobsen et al. 2006; Kjeldsen et al. 2007, 2010). There was, however, one exception, i.e., a group of four strains obtained directly or indirectly with formate as electron donor. These moderately halophilic SRB, unexpectedly, were identified as members of the genus *Desulfonatronovibrio*—a typical representative of alkaliphilic SRB found previously exclusively in soda lakes (Zhilina et al. 1997; Sorokin et al. 2011).

In this report, we describe the properties of a novel halophilic member within the genus *Desulfonatronovibrio*, which is proposed to form a novel species *D. halophilus*.

## Methods

### Samples

Sediment samples of the top 10 cm layer were obtained in July 2010 from four hypersaline chloride–sulfate lakes located in the southern part of the Kulunda Steppe, south-western Siberia (Altai, Russia). The lake brines had a pH range of 7.6–8.2, a salinity from 125 to 340 g l^−1^, a sulfate content from 0.13 to 1.4 M and an acid labile sulfides (FeS + HS^−^) content from 0.3 to 17 mM. The individual sediments were mixed in equal proportions to make a single inoculum used to enrich for halophilic SRB.

### Enrichment and cultivation

Enrichment and routine cultivation of halophilic SRB was performed at 28 °C on a mineral medium containing 2 M NaCl buffered with 0.1 M HEPES at pH 7.5 as described previously (Sorokin et al. 2012). The pH dependence was examined at Na^+^ content of 1 M, using the following filter-sterilized buffers: for pH 6–8, 0.1 M HEPES and NaCl/NaHCO_3_; for pH 8.5–10, a mixture of sodium bicarbonate/sodium carbonate containing 0.1 M NaCl. To study the influence of salt concentration on growth, the HEPES-buffered media with pH 7.5 containing 0.2 and 4 M NaCl were mixed in different proportions.

### Analytical procedures

Sulfide was measured colorimetrically (Trüper and Schlegel 1964) after precipitation in 10 % (w/v) Zn acetate. Cell protein was determined according to Lowry et al. (1951) after removal of sulfide/thiosulfate and washing the cell pellet several times with 2 M NaCl containing 0.05 M HCl. Phase contrast microphotographs were obtained with a Zeiss Axioplan Imaging 2 microscope (Göttingen, Germany). For electron microscopy, the cells were negatively contrasted with 1 % (w/v) uranyl acetate containing 0.5 M NaCl and observed in JEOL 100 (Japan) transmission electron microscope. Polar lipids were extracted from 50 mg of freeze-dried cells with acidic methanol and the fatty acid methyl esters were analyzed by GC–MS according to Zhilina et al. (1997).

### Genetic and phylogenetic analysis

The isolation of the DNA and determination of the G + C content of the DNA were performed according to Marmur (1961) and Marmur and Doty (1962), respectively. For molecular analysis, the DNA was extracted from the cells using lysis in 1 % (wt/vol) SDS/0.2 M NaOH at 60 °C and purified with the Wizard Preps Kit (Promega, USA). The nearly complete 16S rRNA gene was obtained using general bacterial PCR primers 11f and 1492r (Lane 1991). The sequences were aligned with sequences from the GenBank using CLUSTAL W and the phylogenetic tree was reconstructed using either the neighbor-joining or maximum likelihood algorithms in the TREECONW program package (van de Peer and de Wachter 1994). The full dsrAB gene was amplified using the primers DSR-1Fmix and DSR-4Rmix followed by agarose purification according to Miletto et al. (2008). The sequences were aligned using Codoncode aligner (CodonCode Corp., Dedham USA) software. The phylogenetic analysis was performed using ARB software (Ludwig et al. 2004). Sequences were aligned with complete length sequences of closest relatives from the order *Desulfovibrionales* obtained from updated *dsr*AB database (Loy et al. 2009) using the ARB FastAligner utility. The maximum likely hood method, RAxML (implemented in ARB), was used to calculate the resulting phylogenetic tree.

## Results and discussion

### Enrichment and isolation of pure cultures

**Fig. 1:**
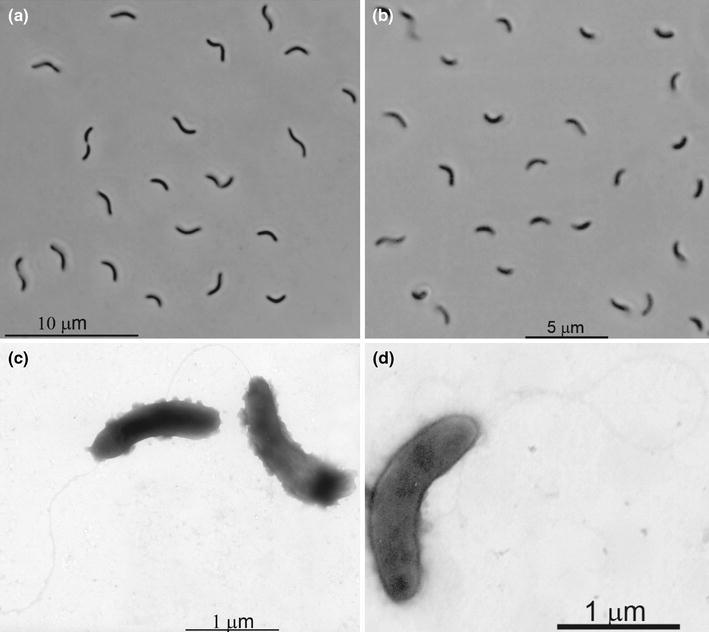
Cell morphology of strains HTR1 (**a**, **c**) and HTR6 (**b**, **d**) grown at pH 8 and 1 M NaCl with formate and thiosulfate. **a**, **b** phase contrast microphotographs, **c**, **d** electron microphotograph of positively stained cells

Four positive anaerobic sulfidogenic enrichment cultures with either formate or ethanol as electron donor and sulfate or thiosulfate as electron acceptor at 2 M NaCl resulted in a domination of motile vibrio-shaped SRB (Fig. 1), which were further purified on the medium with formate by serial dilutions to extinction. Thus, four SRB strains were obtained designated HTR1, HTR6, HTR9 and HTR10. Strains HTR1 and HTR10 were obtained from an enrichment on EtOH + thiosulfate followed by purification on formate + thiosulfate; strain HTR6 was obtained from a direct enrichment with formate and sulfate and strain HTR9 from an enrichment on ETOH + sulfate followed by purification on formate + sulfate. The purity of the isolates was evident from the uniform cell morphology and from 16S rRNA gene sequencing.

### Identification

**Fig. 2:**
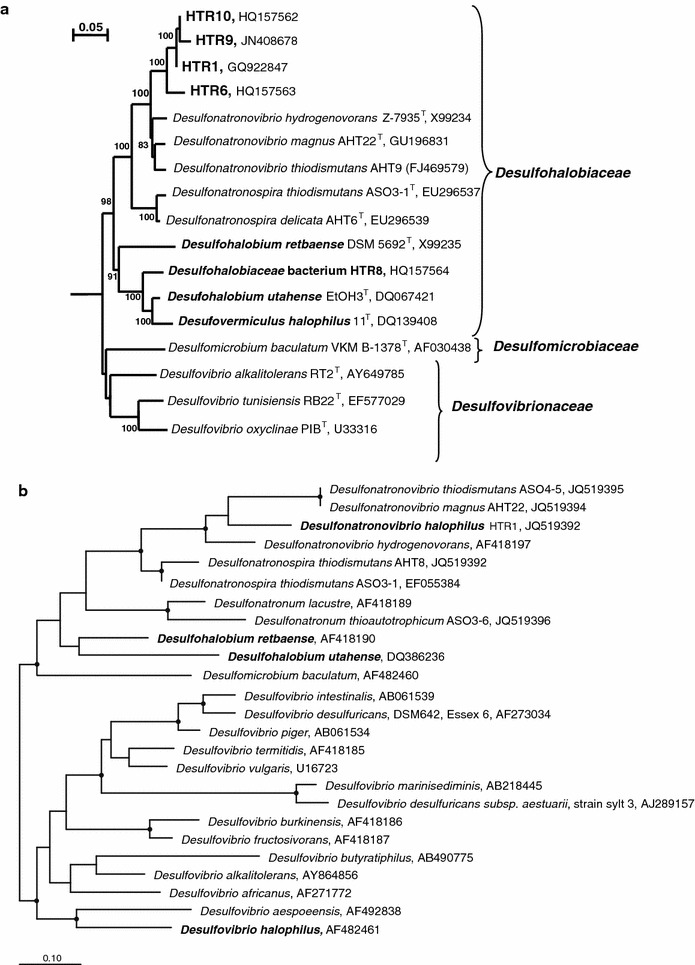
Phylogenetic position of HTR strains within the order *Desulfovibrionales* based on 16S rRNA gene (**a**) and *dsr*AB gene (**b**) sequence analysis. Tree 16S rRNA gene tree topography and evolutionary distances are obtained by the neighbor-joining method with Jukes and Cantor distances. The *numbers on the nodes* indicate bootstrap values above 80 %. The halophilic representatives are in *bold*. The *dsr*AB gene-based tree topography and evolutionary distances are obtained by the Maximum Likelihood method, RAxML using 250 round of bootstrap (≥90 % is indicated by a s*olid dot*) and species AF418190 as a filter. In total 769 positions were used for calculation. All halophilic representatives are in *bold*

Phylogenetic analysis based on 16S rRNA gene sequencing showed that the HTR strains were closely related to each other (sequence similarity 97–99 %) and that they formed a novel cluster within the genus *Desulfonatronovibrio* (94–96 % sequence similarity to the 3 described species), which is a member of the family *Desulfohalobiaceae*, order *Desulfovibrionales* (Kuever et al. 2005) (Fig. 2a). From the four strains, HTR1, HTR9 and HTR10 were more closely related and HTR6 was somewhat more distant, although the phenotype of the isolates showed no obvious difference (see below). Phylogenetic analysis of the functional marker of SRB, the *dsr*AB gene, of strain HTR1 confirmed its affiliation to the type species of the genus *Desulfonatronovibrio*, *D. hydrogenovorans* (82 % nucleotide sequence similarity and 91 % protein sequence similarity). However, the sequence similarity level of both markers indicated that the novel halophilic isolates belonged to a new species.

The analysis of cellular fatty acids in polar lipids of strains HTR1 and HTR6 showed highly similar profiles with a domination of saturated C16:0 and C18:0. Furthermore, a general similarity to the profiles of the previously described members of the genus *Desulfonatronovibrio* was also evident (Supplementary Table S1). However, a direct comparison may not be possible, since there was at least 2 pH units difference during the growth of halophiles and alkaliphiles.

### Physiology

All HTR strains had a limited anaerobic respiratory metabolism typical for the genus *Desulfonatronovibrio*, restricted to utilization of H_2_, formate and pyruvate as electron donors with sulfate, thiosulfate and sulfite as electron acceptors. In contrast to the alkaliphilic species *D. thiodismutans* and *D. magnus*, but similar to the type species *D. hydrogenovorans*, the halophilic isolates were unable to grow by thiosulfate or sulfite dismutation. Growth with H_2_ and formate was only possible in the presence of acetate (i.e. lithoheterotrophically). Fermentative growth with pyruvate was not observed.

### Influence of pH and sodium on growth and activity of strain HTR1

**Fig. 3:**
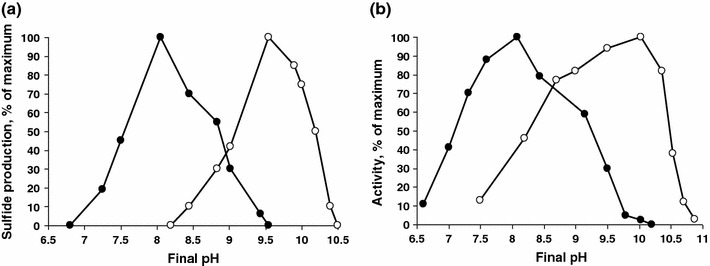
Influence of pH at 1 M NaCl on growth (**a**) and sulfidogenic activity of resting cells (**b**) of strain HTR1 from salt lakes in comparison with *Desulfonatronovibrio thiosulfatophilum* AHT9 from soda lakes (grown at 0.6 M Na^+^). Both organisms were grown with formate/acetate + thiosulfate and the washed cells were tested with formate + thiosulfate. *Closed circles* HTR1, *open circles* AHT9. The results represent average values obtained in from 2 duplicates

**Fig. 4:**
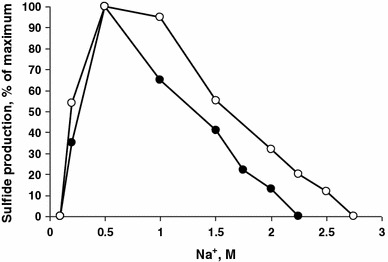
Influence of NaCl at pH 8 on growth (*closed circles*) and activity of washed cells (*open circles*) of strain HTR1 with formate as *e*-donor and thiosulfate as *e*-acceptor. The results represent average values obtained from 2 duplicates

Strain HTR1 grew optimally in NaCl brines at pH 8, thus being a halophile, in contrast to the known obligately alkaliphilic representatives of the genus *Desulfonatronovibrio* from soda lakes. The pH range for growth was from 7.2 to 9.4 (Fig. 3a) which qualified HTR1 as an alkalitolerant halophile. Washed cells were active in a slightly wider pH range, but the sulfidogenic activity was almost completely inhibited at pH 10 in a soda buffer optimal for the soda lake SRB (Fig. 3b). At an optimal pH of 8, strain HTR1 grew at NaCl concentrations from 0.2 to 2 M with an optimum at 0.5 M, and washed cells produced sulfide up to 2.5 M NaCl (Fig. 4). According to these data strain HTR1 is a moderate halophile.

Overall, the four halophilic lithotrophic SRB strains isolated from sediments of Siberian hypersaline salt lakes represent a novel, compact, phylogenetic group within the genus *Desulfonatronovibrio* clearly differentiating from its recognized species by the neutral pH optimum and inability to grow above pH 9.4 (Table 1). We propose to assign these strains into a new species *Desulfonatronovibrio halophilus*.

**Table 1:** Phenotypic characteristics of the halophilic strain HTR1^T^ in comparison with the haloalkaliphilic representatives of the genus *Desulfonatronovibrio*

Property	HTR1^T^	*Dsnv. hydrogenovorans* ^a^	*Dnsv. thiodismutans* ^b^	*Dnsv. magnus* ^b^
Cell width (μm)	0.5	0.5	0.3–0.4	0.6–0.8
Autotrophy	−	−	±	−
Fermentation and oxidation of pyruvate	+	−	+	+
Growth by dismutation of thiosulfate or sulfite	−	Not shown	Thiosulfate, sulfite	Thiosulfate, sulfite
pH range (optimum)	7.2–9.4 (8.0)	8.0–10.2 (9.6)	8.5–10.5 (9.5–10)	8.5–10.5 (10)
Maximal salt tolerance	2.0	2.0	2.0–3.0	2.0
Predominant PLFA	16:0, 17:0, 18:0	16:0, 18:0	18:0, 18:1ω7c	i15:0, ai15:0,
				i16:0, i17:1ω8
G + C (mol%)	45.7	48.6	41.8–42.9	43.0

^a^Zhilina et al. (1997)

^b^Sorokin et al. (2011)

### Electronic supplementary material

Below is the link to the electronic supplementary material.

Supplementary material 1 (PDF 62 kb)

## Appendix

### Description of *Desulfonatronovibrio halophilus* sp. nov.

(ha.lo’phi.lus Gr. n. *hals* salt; Gr. adj *philos*, loving; N.L. masc. adj. *halophilus* salt-loving, halophilic).

Cells are Gram-negative, vibrio-shaped, 0.4 × 1–2 μm, and motile by a single polar flagellum. Strictly anaerobic and respiratory, using hydrogen, formate and pyruvate as *e*-donor and sulfate, thiosulfate and sulfite as *e*-acceptor. Pyruvate, thiosulfate and sulfite are not dismutated. Alkalitolerant with a pH range for growth between 7.2 and 9.4 and an optimum around 8 and moderately halophilic with a range from 0.2 to 2.0 M NaCl (optimum at 0.5 M). Mesophilic, with a maximum temperature for growth at 45 °C and an optimum at 37 °C. The predominant fatty acids in the polar membrane lipids include 16:0, 18:0 and 18:1ω7c. The G + C content of the genomic DNA of strain HTR1 is 45.7 mol% (*T*_m_). The type strain is HTR1^T^ (DSM24312^T^ = UNIQEM U802^T^). Isolated from sediments of soda lakes in south-western Siberia. The GenBank 16S rRNA gene sequence accession number of the type strain is GQ922847.
