# Supplementary material for: Desulfonatronovibrio halophilus sp. nov., a novel moderately halophilic sulfate-reducing bacterium from hypersaline chloride–sulfate lakes in Central Asia
Source: Extremophiles. 2012 Apr 10;16(3):411–7. doi: 10.1007/s00792-012-0440-5 (PMC3346931; doi:10.1007/s00792-012-0440-5)
Supplement: Supplementary file 1 — Supplementary material 1 (PDF 62 kb) [file 792_2012_440_MOESM1_ESM.pdf]

Supplementary TableS1

PLFA profiles (major FA with content above 3%) of halophilic HTR strains in comparison with the type strains of alkaliphilic species of the genus *Desulfonatronovibrio*. Cells were grown at 30°C and harvested in late logarithmic growth phase. The HTR strains were grown at 1 M NaCl, pH 7.5; the soda lake type strains were grown at pH 9.5-10 and 0.5-1.0 M total Na^{+}. Dsnv.hgv. – *Desulfonatronovibrio hydrogenivorans*; Dsnv. thd – *Desulfonatronovibrio thiodismutans*; Dsnv. m – *Desulfonatronovibrio magnus*

FA	**HTR1^{T}**	**HTR6**	Dsnv. hgv	Dsnv. thd	Dsnv. m
14:1	3.2	4.1	5.6		
2OH-14:0				3.9	
i15:0	5.8	7.5	3.7		13.4
ai15:0			3.9		12.8
i16:1					8.4
i16:0	6.5	9.1	4.4	3.7	10.6
16:0	23.1	19.3	32.8		7.3
16:1ω7c	6.8	3.2			
10Me16:0	3.0				
i17:1ω8					10.5
ai17:1ω7					7.3
17cyc					3.1
17:0	12.1	5.8	5.9	6.5	
i18:1ω7					4.1
18:1ω9			3.4		
18:1ω11			5.9		
18:1ω7c	8.5	11.4		15.1	8.6
18:0	15.0	23.3	18.2	44.8	3.0
